# Doppler images of intra-pulmonary shunt within atelectasis in anesthetized children

**DOI:** 10.1186/s13089-016-0055-7

**Published:** 2016-12-01

**Authors:** Cecilia M. Acosta, Gerardo Tusman, Mauro Costantini, Camila Echevarría, Sergio Pollioto, Diego Abrego, Fernando Suarez-Sipmann, Stephan H. Böhm

**Affiliations:** 1 Department of Anesthesia, Hospital Privado de Comunidad, Córdoba 4545, 7600 Mar Del Plata, Buenos Aires, Argentina; 2 Department of Radiology, Hospital Privado de Comunidad, Mar Del Plata, Buenos Aires, Argentina; 3 Department of Pediatric Surgery, Hospital Privado de Comunidad, Mar Del Plata, Buenos Aires, Argentina; 4 Section of Anesthesia and Critical Care Hedenstierna Laboratory, Department of Surgical Sciences, Uppsala University Hospital, Uppsala, Sweden; 5 CIBER de Enfermedades Respiratorias, Instituto de Salud Carlos III, Madrid, Spain; 6 Swisstom AG, Landquart, Switzerland

**Keywords:** Atelectasis, Intra-pulmonary shunt, Children, Lung ultrasound, Recruitment maneuvers

## Abstract

**Background:**

Doppler images of pulmonary vessels in pulmonary diseases associated with subpleural consolidations have been described. Color Doppler easily identifies such vessels within consolidations while spectral Doppler analysis allows the differentiation between pulmonary and bronchial arteries. Thus, Doppler helps in diagnosing the nature of consolidations. To our knowledge, Doppler analysis of pulmonary vessels within anesthesia-induced atelectasis has never been described before. The aim of this case series is to demonstrate the ability of lung ultrasound to detect the shunting of blood within atelectatic lung areas in anesthetized children.

**Findings:**

Three anesthetized and mechanically ventilated children were scanned in the supine position using a high-resolution linear probe of 6–12 MHz. Once subpleural consolidations were detected in the most dependent posterior lung regions, the probe was rotated such that its long axis followed the intercostal space. In this oblique position, color Doppler mapping was performed to detect blood flow within the consolidation. Thereafter, pulsed waved spectral Doppler was applied in the previously identified vessels during a short expiratory pause, which prevented interferences from respiratory motion. Different flow patterns were identified which corresponded to both, pulmonary and bronchial vessels. Finally, a lung recruitment maneuver was performed which leads to the complete resolution of the aforementioned consolidation thereby confirming the pathophysiological entity of anesthesia-induced atelectasis.

**Conclusions:**

Lung ultrasound is a non-invasive imaging tool that not only enables the diagnosis of anesthesia-induced atelectasis in pediatric patients but also analysis of shunting blood within this consolidation.

**Electronic supplementary material:**

The online version of this article (doi:10.1186/s13089-016-0055-7) contains supplementary material, which is available to authorized users.

## Background

Anesthesia-induced atelectasis is a well-known entity observed in approximately 68–100% of pediatric patients undergoing general anesthesia [1–4]. The collapse of dependent lung zones starts with anesthesia induction but can persist for hours after surgery. Anesthesia-related atelectasis have a number of negative clinical consequences such as the impairment of arterial blood oxygenation and lung mechanics [5–7] as well as the predisposition for ventilator-associated lung injury caused by tidal recruitment (i.e., the repetitive opening and closing of unstable lung units during mechanical ventilation) and tidal overdistension of the non-atelectatic regions [8–10].

Lung ultrasound (LUS) has demonstrated its high sensitivity and specificity for diagnosing the entity of anesthesia-induced atelectasis in mechanically ventilated patients [11, 12]. LUS can also reveal tidal recruitment occurring mainly at the boundary of atelectatic lung tissue and its complete resolution after an appropriate lung recruitment maneuver [13].

The use of Doppler for the study of pulmonary vessels within consolidated lung areas has already been reported by several authors [14–16]. Yuan et al. [15] described the role of Doppler in many pulmonary diseases such as infarction, pneumonia, pulmonary sequestration, abscesses and tumors. The same authors described different flow patterns in pulmonary vessels and proposed that such patterns may be helpful in differentiating malignant tumors from benign consolidations such as pneumonias, abscesses or obstructive atelectasis [14]. Using Doppler, Görg et al. [16] described the dual arterial supply within different kinds of consolidations. They discriminated pulmonary from bronchial vessels by the pattern of the spectral flow signal.

Even though it has been known that intra-pulmonary shunting is the main reason for the deterioration of gas exchange in anesthesia-induced atelectasis, the visualization of shunting blood usually requires technologies such as PET, SPECT or arteriography. As opposed to these complex diagnostic tools, the examination of pulmonary vessels within consolidated lung areas by Doppler is simple, non-invasive, non-ionizing and perfectly suitable to assess shunt at the bedside [14, 15]. Furthermore, Doppler is capable of differentiating pulmonary from bronchial vessels within a lung consolidation [16]. However, to our knowledge, Doppler has never before been used to visualize the intra-pulmonary shunting of blood within anesthesia-induced atelectasis. Therefore, the aim of this case series is to provide the first evidence of shunt in anesthetized mechanically ventilated children, in whom high-resolution images of atelectasis can easily be obtained using a linear high-frequency probe.

## Sonographic diagnosis of anesthesia-induced atelectasis in the operating room and evidence of intra-pulmonary shunt

Three pediatric patients aged two months, one year and four years of age undergoing general anesthesia for abdominal laparoscopic surgery were analyzed. A protective ventilation strategy was applied using volume-controlled ventilation with a tidal volume of 6 ml/kg ideal body weight, a positive end-expiratory pressure (PEEP) of 5 cmH_2_O, an inspiration/expiration (I:E) ratio of 1:1 and FIO_2_ of 0.5. Respiratory rate was adjusted to keep end-tidal CO_2_ between 35 and 40 mm Hg.

**Fig. 1:**
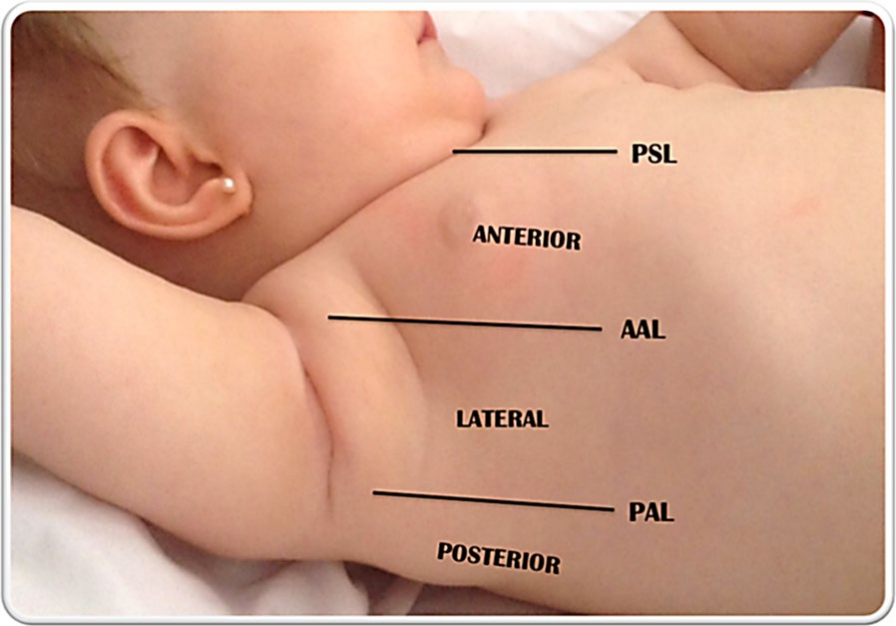
Each hemithorax was divided into three regions: anterior, lateral, and posterior regions corresponding to the anatomical landmarks of the *parasternal line*, *anterior* and *posterior axillary lines*. *AAL* anterior axillary line; *PAL* posterior axillary line and *PSL* parasternal line

LUS assessment was performed with the portable echograph MicroMaxx (Sonosite, Bothell, WA, USA) using a linear probe of 6–12 MHz. Patients were examined in the supine position placing the probe longitudinally as recommended by expert consensus [17]. Given the small size of the chest of neonates or infants, LUS examination of each hemithorax was performed in three regions—anterior, lateral and posterior [18] (Fig. 1).

Subpleural consolidations were identified as atelectasis when they were associated with the following signs [11]: absence of lung sliding and A-lines, presence of multiple spaced B-lines or coalescent B-lines born in subpleural consolidation, static air bronchograms and the presence of a pulse sign. The finding of tidal recruitment within the consolidation reinforced the diagnosis of atelectasis. Finally, the reversal of the consolidation by the recruitment maneuver confirmed the entity of anesthesia-induced atelectasis [13].

Atelectasis should be distinguished from other types of consolidation like pneumonia and pulmonary embolism. Pneumonia appears as a consolidation with irregular and somewhat blurred margins, commonly associated with dynamic tree-shaped air bronchograms and pleural effusion. The sonographic signs of pulmonary embolism consist of generally two or more small pleural based, hypoechoic consolidations with sharp margins and without central vascularization. Importantly, these two kinds of consolidations cannot be reverted by a lung recruitment maneuver.

**Fig. 2:**
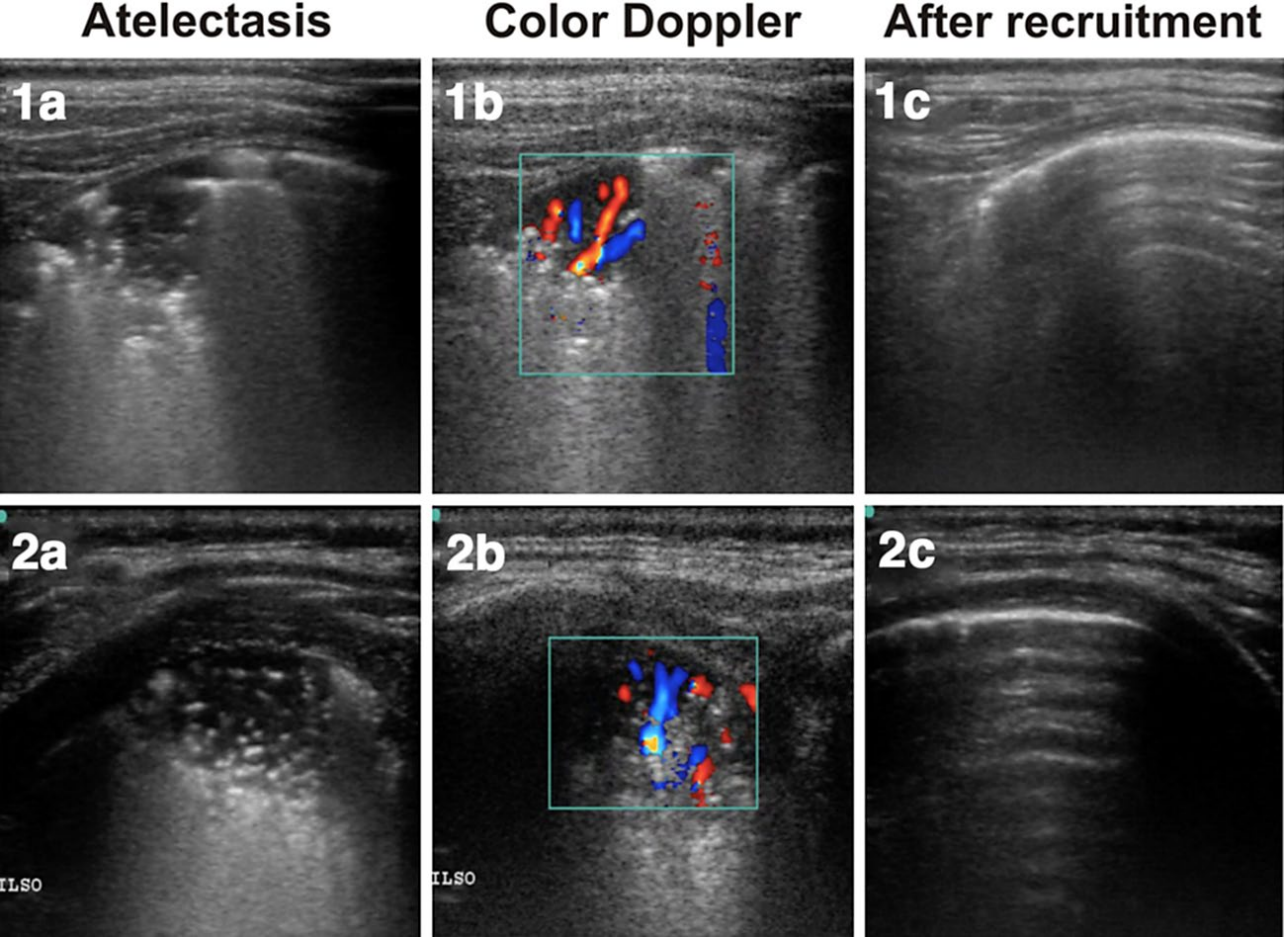
Color Doppler images of intra-pulmonary shunt. The images belong to two mechanically ventilated anesthetized children: (1) 1 year old and (2) 2 months old. **a** Atelectasis is observed as subpleural consolidations with air bronchograms and coalescent B-lines. **b** Color Doppler revealed lung vessels within the atelectasis showing a radial distribution to the lung periphery. **c** Normal lung tissue after successful treatment of the atelectasis by a recruitment maneuver, the normal aeration prevents the pulmonary vessels from being seen by lung sonography. These images correspond to the Additional file 1: Video 1

Once subpleural consolidations were detected in the most dependent lung areas, the probe was rotated to overcome the acoustic shadows of the ribs and an oblique intercostal view was used for further analysis (Fig. 2a, b).

Color Doppler ultrasound detected pulmonary vessels within consolidated lung areas [14–16]. Most vessels showed a radial anatomical configuration (Fig. 2; Additional file 1: Video 1). Later on, pulsed wave spectral Doppler analysis was performed by positioning the sample volume into the center of the lumen of the detected vessel and by placing the ultrasound beam as parallel to the axial flow (≤60°) as possible [14]. To avoid interferences from mechanical ventilation, spectral Doppler analysis was done during an expiratory hold and spectral waveforms of similar shape were sampled from at least five consecutive cardiac cycles.

**Fig. 3:**
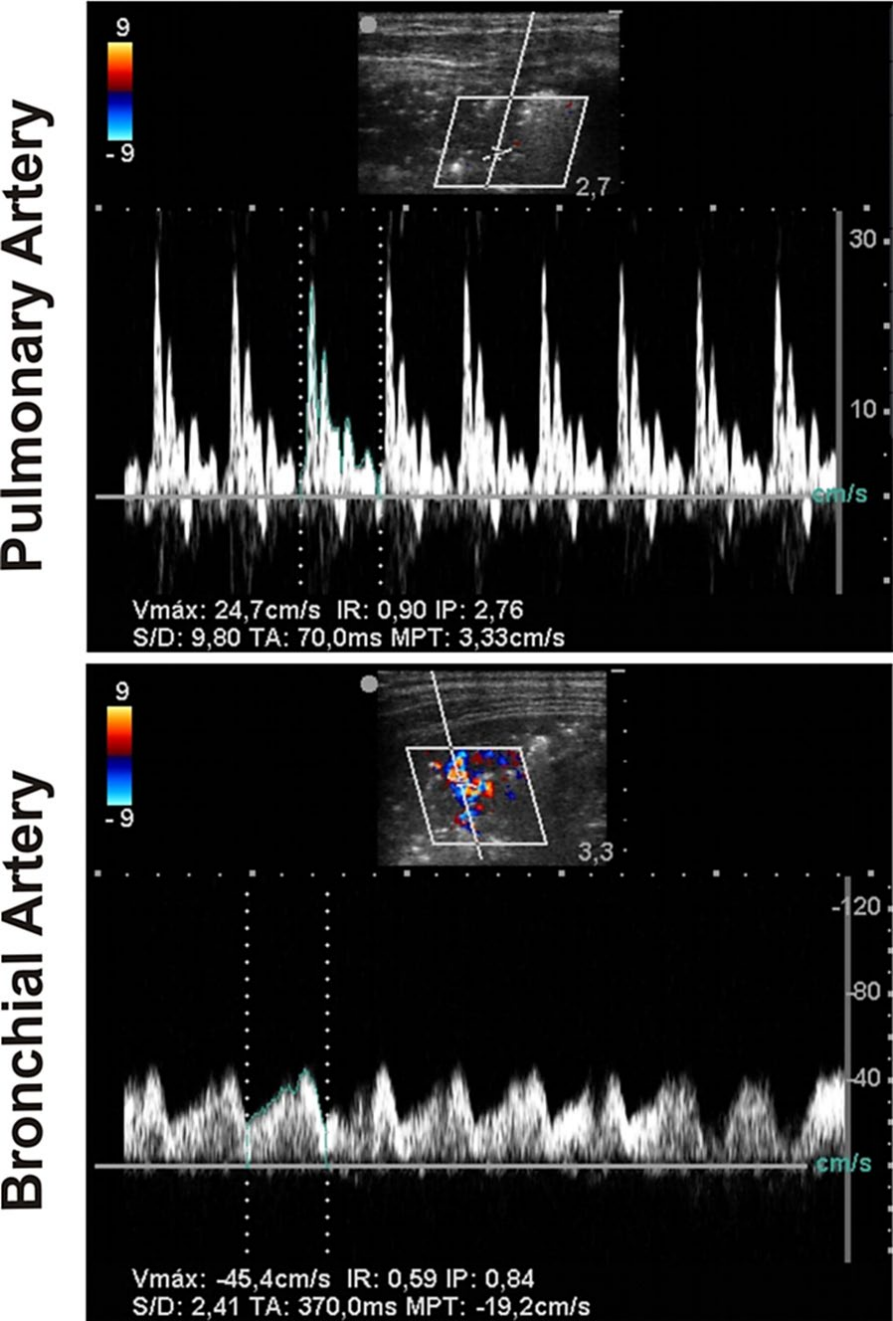
Spectral Doppler analysis of the three types of vessels within the lung. Spectral Doppler analysis of a pulmonary artery (*upper*) and a bronchial artery (*lower*) within a representative anesthesia-induced atelectasis in 4-year-old patient. Spectral Doppler was performed during a brief expiratory pause. Pulmonary artery showed lower Doppler maximal velocity and higher resistance (IR) and pulsatile (IP) indexes than the bronchial artery presumably due to hypoxic pulmonary vasoconstriction

We observed two different arterial flow patterns within atelectatic areas (Fig. 3):

- A high-impedance flow signal. This flow pattern originates from a pulmonary artery because as activation of the local hypoxic pulmonary vasoconstriction reflex results in high impedance [19].
- A low-impedance monophasic flow signal that corresponded to bronchial arteries [19].

Finally, we performed a lung recruitment maneuver as previously described [13, 20]. The maneuver consisted of a brief and controlled step-wise increase in airway pressure aiming at re-expanding the atelectasis. The maneuver started from 5 cmH_2_O of PEEP that was then increased in 5 cmH_2_O steps until airway opening pressure of 30 cmH_2_0 was reached. At this point, LUS images confirmed the resolution of atelectasis, with the subsequent improvement in lung aeration. Thereafter, a step-wise decrease in PEEP allowed the detection of the minimum level that prevented the reappearance of atelectasis seen in the LUS images. The complete resolution of dependent lung atelectasis by the recruitment maneuver was confirmed 5 min after this maneuver by longitudinal and oblique LUS examination in three regions in each hemithorax, anterior, lateral y posterior; leading to the diagnosis of anesthesia-induced atelectasis [4] (Fig. 2; Additional file 1: Video 1). The normally aerated lung tissue now reflected the ultrasound beam such that intra-pulmonary vessels were no longer detectable.

## Commentary

In this case series, we are presenting Doppler images of blood shunting within anesthesia-induced atelectasis in mechanically ventilated children. Doppler analysis of these lung vessels was similar to the one already described for different types of lung consolidations.

This finding has two important implications: (1) *academic/educational*, since we are not aware of any previous study that presented Doppler images of shunt within consolidated lungs of anesthetized children; (2) *clinical*, which is related to the potential analysis of blood flow in vessels within lung consolidations. Thus, color and spectral Doppler confirm the existence of shunt while the Doppler flow pattern could provide a general idea about the vascular origin of those vessels (arterial vs. venous–bronchial vs. pulmonary). The spectral Doppler analysis of pulmonary vessels could be useful to assess the impact of hypoxic pulmonary vasoconstriction on arterial oxygenation or right ventricular afterload. The role of Doppler analysis of pulmonary and bronchial flow within atelectasis should be evaluated in new protocols.

Therefore, many questions arise: are these regional changes in vascular flow representative of the blood flow pattern within the main pulmonary arteries? Do atelectasis have negative effects on right heart function? Can blood flow throughout the shunt pathways be manipulated by vasoactive drugs? Surely, future studies should be conducted to answer these and many other related questions and to determine the potential clinical value of the presented Doppler-based shunt analysis.

Most published evidence was obtained in spontaneously breathing adults and is thus directly related neither to mechanically ventilated nor pediatric patients. Recently, Mongodi et al. [21] published color Doppler images of vessels within consolidated lung areas of mechanically ventilated critically ill adults and Yekeler et al. [22] presented similar images in spontaneously breathing children suffering from pneumonia. While our findings are in line with those of the latter publications, the demonstration of shunting blood within the atelectasis of mechanically ventilated children with previous healthy lungs is novel and unique.

## Conclusions

Lung ultrasound can easily detect the presence of anesthesia-induced atelectasis and Doppler shunting within them in pediatric patients. Beyond the effects on gas exchange, the clinical impact of this shunting on pulmonary circulation must be analyzed in future studies.

## Additional file

**Additional file 1.** The video belong to two mechanically ventilated anesthetized children. **1** 1 year old and **2** 2 months old. **A** Atelectasis are observed as subpleural consolidation with air bronchograms and coalescents B-lines. **B** Color Doppler revealed lung vessels within the atelectasis showing a radial distribution to the lung periphery. **C** Normal lung tissue after successful treatment of the atelectasis by a recruitment maneuver, the normal aeration prevents the pulmonary vessels from being seen by lung sonography.
